# Fisheries conservation on the high seas: linking conservation physiology and fisheries ecology for the management of large pelagic fishes

**DOI:** 10.1093/conphys/cov059

**Published:** 2016-01-13

**Authors:** Andrij Z. Horodysky, Steven J. Cooke, John E. Graves, Richard W. Brill

**Affiliations:** 1 Department of Marine and Environmental Science, Hampton University, 100 East Queen Street, Hampton, VA 23668, USA; 2 Fish Ecology and Conservation Physiology Laboratory, Department of Biology and Institute of Environmental Science, Carleton University, 1125 Colonel By Drive, Ottawa, ON, Canada K1S 5B6; 3 Department of Fisheries Science, Virginia Institute of Marine Science, College of William & Mary, Gloucester Point, VA 23062, USA; 4 Behavioral Ecology Branch, James J. Howard Marine Sciences Laboratory, Northeast Fisheries Science Center, National Marine Fisheries Service, NOAA, Highlands, NJ 07732, USA

**Keywords:** Bycatch, cardiorespiratory, Fry paradigm, pelagic fishes, post-release survival

## Abstract

Populations of tunas, billfishes and pelagic sharks are fished at or over capacity in many regions of the world. They are captured by directed commercial and recreational fisheries (the latter of which often promote catch and release) or as incidental catch or bycatch in commercial fisheries. Population assessments of pelagic fishes typically incorporate catch-per-unit-effort time-series data from commercial and recreational fisheries; however, there have been notable changes in target species, areas fished and depth-specific gear deployments over the years that may have affected catchability. Some regional fisheries management organizations take into account the effects of time- and area-specific changes in the behaviours of fish and fishers, as well as fishing gear, to standardize catch-per-unit-effort indices and refine population estimates. However, estimates of changes in stock size over time may be very sensitive to underlying assumptions of the effects of oceanographic conditions and prey distribution on the horizontal and vertical movement patterns and distribution of pelagic fishes. Effective management and successful conservation of pelagic fishes requires a mechanistic understanding of their physiological and behavioural responses to environmental variability, potential for interaction with commercial and recreational fishing gear, and the capture process. The interdisciplinary field of conservation physiology can provide insights into pelagic fish demography and ecology (including environmental relationships and interspecific interactions) by uniting the complementary expertise and skills of fish physiologists and fisheries scientists. The iterative testing by one discipline of hypotheses generated by the other can span the fundamental–applied science continuum, leading to the development of robust insights supporting informed management. The resulting species-specific understanding of physiological abilities and tolerances can help to improve stock assessments, develop effective bycatch-reduction strategies, predict rates of post-release mortality, and forecast the population effects of environmental change. In this synthesis, we review several examples of these interdisciplinary collaborations that currently benefit pelagic fisheries management.

## Abstract (precis)

Physiological tools provide a mechanistic basis for understanding fundamental and applied ecology of tunas, billfishes, and pelagic sharks. In this synthesis, we review several templates for the interdisciplinary interactions between physiologists and fisheries scientists and highlight three areas of successful collaborations that directly benefit pelagic fisheries management.

## Introduction

**Figure 1:**
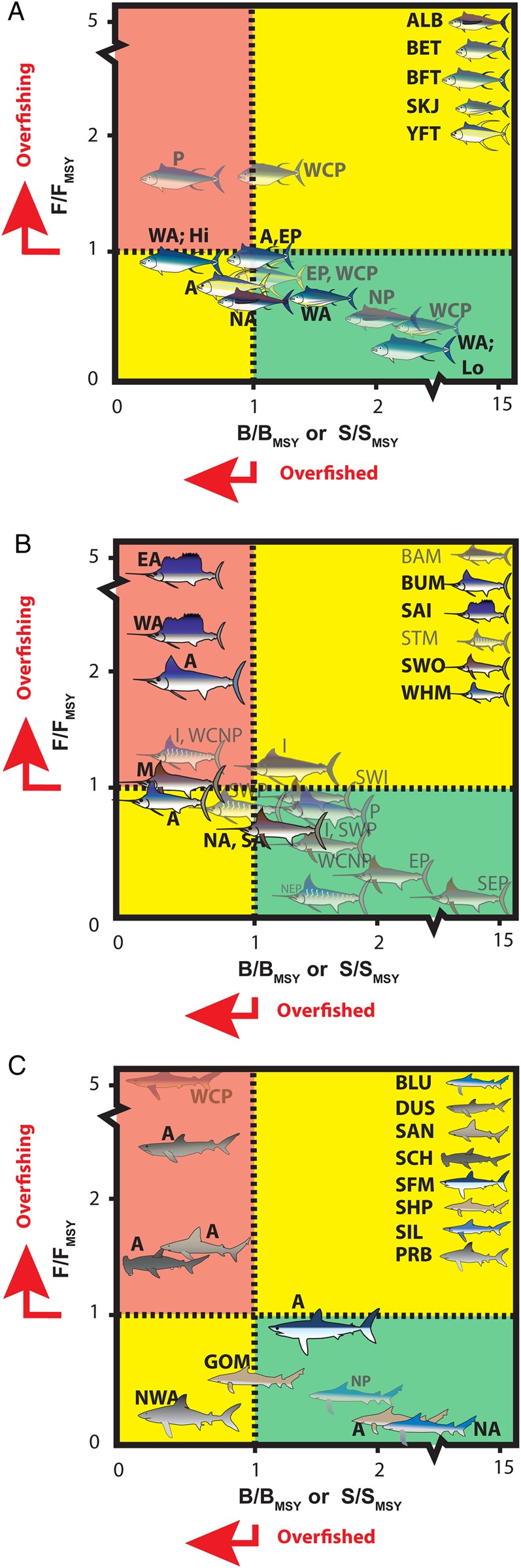
Kobe plots of the current stock status of pelagic fishes, including tunas (**A**), billfishes (**B**) and pelagic sharks (**C**). A species is overfished when biomass (*B*) or stock size (*S*) is less than that at maximal sustainable yield (MSY; i.e. *B* < *B*_MSY_), and overfishing occurs when current fishing mortality (*F*) is greater than that at MSY (i.e. *F* > *F*_MSY_). Red quadrants indicate that a species is overfished and overfishing is occurring, yellow quadrants indicate that a species is either overfished or overfishing is occurring, and species within the green quadrant are neither overfished nor experiencing overfishing. Atlantic species are presented in the foreground of figure. Regional stock abbreviations are as follows: Atlantic (A) [East Atlantic (EA), West Atlantic (WA), North Atlantic (NA), Northwest Atlantic (NW), and South Atlantic (SA)]; Gulf of Mexico (GOM); Mediterranean Sea (M); Indian Ocean (I), and Southwest Indian Ocean (SWI); and Pacific Ocean (P) [East Pacific (EP), Northeast Pacific (NEP), Southeast Pacific (SEP), Southwest Pacific (SWP), and West Central North Pacific (WCNP)]. Abbreviations for tunas (ISSF, 2013) are as follows: albacore (*Thunnus alalunga*; ALB), bigeye tuna (*Thunnus obesus*; BET), Northern bluefin tuna (*Thunnus thynnus*; BFT), skipjack tuna (*Katsuwomis pelamis*; SKJ), and yellowfin tuna (*Thunnus albacares*; YFT). For bluefin tuna, ‘Hi’ and ‘Lo’ refer to high- and low-recruitment scenarios, respectively. Abbreviations for billfishes (NOAA HMS SAFE, 2014; Punt *et al*., 2015) are as follows: black marlin (*Istiompax indica*; BAM), blue marlin (*Makaira nigricans*; BUM), sailfish (*Istiophorus platypterus*; SAI), striped marlin (*Kajikia audax*; STM), swordfish (*Xiphias gladius*; SWO), and white marlin (*Kajikia albida*; WHM). Abbreviations for pelagic sharks (Dulvy *et al*., 2008; NOAA HMS SAFE, 2013, 2014) are as follows: blue shark (*Prionace glauca*; BLU), Atlantic porbeagle (*Lamna nasus*; PRB), shortfin mako (*Isurus oxyrhinchus*; SFM), and silky shark (SIL; ICCAT, 2012; NOAA HMS SAFE, 2013, 2014). For comparison, data for several coastal sharks are included, as follows: dusky shark (*Carcharhinus obscurus*; DUS), sandbar shark (*Carcharhinus plumbeus*; SAN), scalloped hammerhead (*Sphyrna lewini*; SCH), and Atlantic sharpnose shark (*Rhizoprionodon terraenovae*; SHP; (NOAA HMS SAFE, 2013, 2014).

Stocks of many large, highly migratory pelagic fishes (including billfishes, tunas and pelagic sharks; Lesser Antilles Pelagic Ecosystem (LAPE) project) are being harvested at or over capacity (Worm *et al*., 2009), as fisheries fish through food webs to meet the protein demands for a burgeoning human population (Essington *et al*., 2006). The major source of fishing mortality for tunas, billfishes and pelagic sharks is either as targeted catch or bycatch in commercial pelagic longlines, gill nets and purse seines (Dulvy *et al.*, 2008; Graves *et al*., 2012; Jordan *et al*., 2013; Graves and Horodysky, 2015; Oliver *et al*., 2015). Tunas, billfishes and pelagic sharks also support lucrative directed recreational fisheries that often promote catch and release. Commercially targeted populations, including tunas (*Thunnus* spp.; Fig. 1A) and swordfish (*Xiphias gladius*; Fig. 1B), are mostly fished near capacity; many of the bycatch species, such as istiophorid billfishes (Fig. 1B) and sharks (Fig. 1C), are overfished and/or experiencing overfishing, particularly in the Atlantic Ocean (Dulvy *et al*., 2008; Worm *et al*., 2013; NOAA, 2014, 2015; Punt *et al*., 2015).

The heavy exploitation of large pelagic fishes by commercial fisheries necessitates an accurate understanding of the status of their stocks (Uozumi, 2003; Braccini, 2015; Punt *et al*., 2015). Stock assessment models of pelagic fishes typically incorporate catch-per-unit-effort (CPUE) time-series data from commercial fisheries, but there have been notable changes in targeted species, areas fished and depth-specific gear deployments over the years (Lynch *et al*., 2011). For example, in the 1970s pelagic longline gear deployments shifted from shallow sets primarily targeting yellowfin tuna (*Thunnus albacares*) to deeper sets primarily targeting swordfish and bigeye tuna (*Thunnus obesus*; Lynch *et al*., 2012); how these shifts in fishers' behaviours altered the catchability and estimated abundances of target and bycatch species is less clear (Horodysky *et al*., 2007). Attempts to standardize CPUE time series for changes in target species, gear, and spatial behaviour have typically incorporated generalized linear models for Atlantic Ocean species or populations and led to the development of habitat-based standardization (HBS) approaches for population assessments of those in the Pacific Ocean (Lynch *et al*., 2011). In a generalized linear model, environmental data are considered indirectly via the inclusion of variables related to longline gear configurations as fixed effects that serve as proxies for habitats. In contrast, the HBS approach directly incorporates data on oceanographic conditions, fisheries gear behaviour and the ecophysiology and behaviour of pelagic fishes (Hinton and Nakano, 1996; Lynch *et al*., 2012). Finally, although they are not strictly stock assessment models, the recent development of spatial ecosystem, species distribution and seascape spatial distribution models also show great potential to refine spatial understanding of fish behaviour, movements, and distribution (Lehodey *et al*., 2008, 2015; Kearney and Porter, 2009; Alvarez-Berastegui *et al*., 2014; Everson *et al*., 2015).

Collectively, modern assessment and spatial approaches that incorporate environmental variation into pelagic fish stock assessments improve population and distribution estimates. We posit, as have others previously (e.g. Barkley *et al*., 1978; Brill, 1994; Brill and Lutcavage, 2001), that such approaches require a thorough mechanistic understanding of the relationship of fisheries resources to environmental variation (including perturbations of anthropogenic origin) over space and time. Likewise, a better understanding of the lethal and sublethal effects of interactions with gear and handling procedures prior to release is required to improve estimates of total fisheries mortality and the applicability of model outputs. The discipline of conservation physiology can provide these fundamental mechanistic links, because physiology is the transfer function that links specific environmental conditions to behaviour and fitness (Weissburg and Browman, 2005; Jusup *et al.*, 2011; Horodysky *et al*., 2015). However, a rigorous mechanistic understanding of individual physiological response to a specific environmental parameter conducted in a reductionist laboratory setting is a grand challenge for pelagic fishes. Large, rare-event, high-oxygen-demand pelagic species are expensive and difficult to obtain, and in some cases impossible to maintain, in captivity (Box 1). Through interdisciplinary collaboration, these hurdles are being overcome.

**Box 1: Unique attributes of pelagic fishes, and the challenge in studying them**

Large, highly migratory pelagic fishes, herein defined as tunas (Thunnidae), billfishes (Istiophoridae), and pelagic sharks (including Alopidae, Lamnidae, and some Carcharhinidae; Dulvy et al., 2008), inhabit the vast geographical expanse of world's open oceans. During movements and migrations throughout their extremely broad ranges, these fishes interact with tremendous acute and chronic variation in the water column's physicochemical parameters. Collectively, these fishes contain a suite of morphological and physiological adaptations for life in perpetual motion that has expanded their vertical and horizontal niches, including the following: streamlining of body and fins; regional or whole-body endothermy; large gill surface areas; blood with a higher affinity for O_2_; and modifications to cardiac calcium-cycling processes (Brill and Lutcavage, 2001; Bernal *et al*., 2009). Additionally, controlling for phylogeny, these fishes have some of the highest energetic demands and fastest growth rates among fishes (Brill and Bushnell, 1991; Brill, 1996).

The ecophysiology of pelagic fishes is thus a fruitful platform to relate form to function and the environment; however, it remains a frontier field because of the logistical challenges of working with these fishes. Pelagic fishes are generally very patchily distributed in the vast ocean; therefore, obtaining samples is a difficult, time-consuming and very expensive proposition. This is especially true if live animals (or living tissues from them) are required, because the large body sizes and high oxygen demands of these fishes make them extremely challenging to safely control, possess and successfully maintain at sea. Many of these species are thus also exceedingly difficult to transport to, and maintain in, captivity. There are only a few research facilities in the world with the proximity to pelagic habitats and the logistical resources to maintain these fishes; even then, billfishes and many species of pelagic sharks have eluded successful captive husbandry.

Where there are challenges, however, lie opportunities. New technologies inspire new directions. With the continuing evolution of electronic tags, laptop and hand-held computers and tablets, and portable blood analysers, interdisciplinary physiological studies are increasingly moving between the laboratory and ship, each erecting and assessing hypotheses generated in the other. Conservation physiology of pelagic fishes thus represents a rapidly expanding interdisciplinary growth area for the fields of physiology, conservation, and fisheries science, replete with profound socioeconomic and management implications.

Interdisciplinary collaborations between the mechanistically driven physiological sciences, the pattern-oriented behavioural sciences, and the quantitatively driven applied fisheries sciences will greatly advance the synoptic understanding of the environment–pelagic fish–ecosystem interface. An earlier synthesis addressed interactions between mechanistic physiology and field-based and quantitative ecological sciences in the service of the interdisciplinary field of marine and freshwater fisheries science (Horodysky *et al.*, 2015). Here, we examine how physiological studies of large, highly migratory pelagic fishes have improved their management and conservation via interdisciplinary collaborations that are directed at: (i) fish–environment relationships; (ii) bycatch reduction; and (iii) post-release survival.

## Relationships of pelagic fishes to the environment: paradigms

How pelagic fishes relate to their environment bears clear implications for the generation of accurate population assessments and the resultant management and policy decisions. Pelagic fishes sample the environment with sensory receptors tuned to solutes, gases, temperature, bulk flow, electrical and magnetic fields, light, and acoustic vibrations (e.g. Kapoor and Hara, 2001; Sloman *et al*., 2006; Hara and Zielinski, 2007). The distributions and functional characteristics of these receptors are shaped by intense selective pressures according to species-specific life histories and ecologies (e.g. Ladich *et al*., 2006; Horodysky *et al*., 2010; Kaijura *et al*., 2010; Kalinoski *et al*., 2014). Environmental signals amplify to individual behaviour (via physiological abilities and tolerances), from the individual to the population (via behavioural iteration across individuals), and ultimately, from populations to ecosystems (via ecological iteration across species; Weissburg and Browman, 2005; Seebacher and Franklin, 2012). Disruptions from optimal conditions lead to departures from homeostasis, decreasing fitness by negatively affecting survivorship, growth and/or reproduction. Fishes respond to such deviations via complex interactive biochemical, neurological, endocrine and behavioural feedbacks (*sensu* Ricklefs and Wikelski, 2002). The interplay between the sensory, neural, and motor systems renders environmental conditions actionable at the organismal level.

Fry (1947) developed a classic paradigm for the fish–environment interface along different scales of biological organization by elucidating how environmental variation affects individual metabolic scope (defined as the difference between standard and maximal metabolic rate, within which all bioenergetic requirements must be met). Recent applications of aerobic scope modelling unite physiological experiments with spatial and quantitative modelling approaches to predict the effects of environmental change, and demonstrate great potential for improving mechanistic prediction of pelagic fish movements (e.g. Lehodey *et al*., 2013; Del Raye and Weng, 2015). Fry's paradigm forms a template for the interdisciplinary integration of pelagic fish physiology and fisheries science (Claireaux and Lefrancois, 2007) and can be used to assess the relationships between aerobic performance and fitness (Pörtner, 2010), spatial ecology (Del Raye and Weng, 2015) or be combined with more detailed quantitative models to assess bioenergetic effects on fitness (Jørgensen and Holt, 2013; Cooke *et al*., 2014a).

Modern quantitative approaches, including the metabolic theory of ecology, dynamic energy budget, species distribution models, and HBS-based assessments, may also provide both mechanistic and quantitative explanations of the organism–environment interaction by modelling energy fluxes and growth potential as a function of environmental conditions (Maury, 2010; Jusup *et al*., 2011; Nisbet *et al*., 2012). They can also be used to assess and predict current and future patterns of distribution and abundance (Hinton and Nakano, 1996; Humston *et al*., 2000; Del Raye and Weng, 2015; Everson *et al*., 2015; Lehodey *et al*., 2015). Collectively, these approaches share a common challenge, namely how to parameterize models with robust data that encapsulate the relevant climatic, temporal, ontogenetic, and intraspecific variation (Cooke *et al*., 2014b). But simply correlating field data with catch data to infer environment–fish relationships is circular reasoning. As discussed by Brill (1994) and Brill and Lutcavage (2001) for tuna fisheries, this is especially true when field-based catch or abundance proxies are used to determine the effects of environmental conditions on catch or abundance. Rather than field correlations alone, laboratory (Blank *et al*., 2004) and/or shipboard investigations (Fritsches *et al*., 2005; Galli *et al*., 2009), combined with field surveys and electronic tagging (e.g. Holland *et al*., 1990, 1992; Josse *et al*., 1998; Brill *et al*., 1999; Bertrand *et al*., 2003; Hussey *et al*., 2015), can improve mechanistic understanding of the dynamic temporal and spatial nature of the fish–environment interface that can be comprehended by stakeholders and effectively applied by resource managers.

The desire for simplicity and dimension reduction in pelagic fish habitat modelling has led to mechanistic missteps that make little sense from the perspective of the fishes being modelled (Brill and Lutcavage, 2001). This is likely to result from the fundamental disconnect between how humans and fishes sample the environment. For various reasons, scientists studying pelagic fishes (often via fishery-dependent means) measure variables of spatiotemporal relevance to humans (geography, depth, and time); these may be of little relevance to fish. In contrast, individual fish can only experience their immediate microhabitat (Helmuth, 2009), stratifying by the physicochemical variables they can detect in their immediate surroundings given their sensory mechanisms (temperature, oxygen, salinity, light and day length, substrate, and prey/predator abundance).

A mechanistic understanding of habitat selection by pelagic fishes therefore considers the following tenets: (i) individuals experience only their immediate surrounding environment (delimited by their multimodal sensory integration abilities in the current physicochemical conditions); and (ii) individuals can only truly prefer an environmental variable they can sense and where there is a direct relationship between receptor and/or afferent nerve activity and the physical variable (Horodysky *et al*., 2015). For these reasons, species-specific depth ‘preference’ mentioned in tagging studies and population assessments of large pelagic fishes (e.g. Block *et al*., 2001; Ward and Myers, 2005; Evans *et al*., 2008) is a mechanistically nonsensical concept; fishes do not have an absolute sense of depth *per se* (or its correlate, for that matter) and thus cannot prefer it (Bernal *et al*., 2009). ‘Depth preference’ is thus more likely to be a result of the interactions of light, temperature, and oxygen conditions from the perspective of a pelagic fish, and may or may not be a useful covariate for modelling fish vertical movements. Strongly positive covariation with the real mechanistic driver of behaviour would result in little bias when using depth as a proxy. However, if depth either does not correlate or has an inverse (or unknown) relationship with a mechanistic driver, model outputs may be completely inaccurate. Regardless, we posit that the term ‘depth preference’ should be avoided altogether in the literature.

A corollary concern which involves the application of HBS standardizations of CPUE data for pelagic fish stock assessments is their underlying assumption that feeding motivation (and thus catchability) is proportional to time spent at depth (Graves *et al*., 2003). This assumption superimposes an unknown and unmeasured behavioural driver on a non-mechanistic covariate to parameterize a population assessment. Feeding motivation integrates prey availability to the predator, energetics, homeostasis, predation risk, and energy expenditure. What if pelagic fishes are more motivated to feed at cooler, dimmer depths rather than in the surface waters where most species spend the majority of time? Habitat-based standardization applications that make this assumption risk mischaracterizing catchability and decoupling catch from abundance (Brill and Lutcavage, 2001; Horodysky *et al*., 2007). Reliable demographic estimates of pelagic fish populations require a rigorous understanding of which (and when) environmental and biological parameters are true drivers that affect the fitness, performance and survival of pelagic fishes, and which (and when) they are noise, (Helmuth, 2009). Such mechanistic understanding has been provided via the study of pelagic fish physiology.

## Mechanistic interdisciplinary investigations link a species' physiology and ecology

Behaviours are often directed by the need to maintain physiological homeostasis in the face of environmental variation, coupling the physiology of a species to its ecology. Several physicochemical variables have received attention as mechanistic drivers of the aerobic scope and behaviour of pelagic fishes. In this section, we describe interdisciplinary collaborations between fish physiologists and pelagic fisheries scientists that have combined laboratory and field-based approaches to examine how pelagic fishes interact with driving variables. Powerful examples of this union include the explanation of the vertical movement patterns of tunas and other pelagic fishes via the effects of temperature on cardiovascular performance (e.g. Brill *et al*., 1998, 1999; Brill and Bushnell, 2001; Blank *et al*., 2004; Galli *et al*., 2009, 2011; Shiels *et al*., 2015), the expansion of vertical habitat enabled by the sensory thermophysiology (Brill *et al*., 2005; Fritsches *et al*., 2005) and the compression of realized niches in pelagic fishes based on dissolved oxygen (Brill, 1994; Prince and Goodyear, 2006; Prince *et al*., 2010).

Temperature controls biochemical reactions and metabolic rates (Fry, 1947) and is arguably the best understood and most influential environmental variable driving pelagic fish behaviour and distribution (Brill, 1994; Dickson, 1995; Braun *et al*., 2015). There has been much study of the thermal physiology and metabolic rates of tunas (reviewed by Graham and Dickson, 2001; Korsmeyer and Dewar, 2001). Inferences from these studies have been extended by analogy to billfishes and some pelagic elasmobranchs. Owing to a suite of anatomical and physiological adaptations, the aerobic scope, standard metabolic rate, and maximal aerobic metabolic rate of tunas are each three to five times greater than those of active teleosts and are dependent on environmental temperatures (Brill and Bushnell, 1991; Dewar and Graham, 1994; Brill, 1996; Korsmeyer and Dewar, 2001). The vertical and horizontal extent of habitat, combined with wide geographical distribution of highly migratory pelagic species, exposes them to a wide range of ambient temperatures. Recent studies have expanded this synthesis further by examining the effect of increased global temperatures and climate change on pelagic fish reproduction and distribution (Polovina, 2007; Muhling *et al*., 2011; Bell *et al*., 2013; Lehodey *et al*., 2013, 2015).

In order to exploit vertical and horizontal thermal gradients better across large and potentially shifting ranges, a phylogenetically diverse group of pelagic fishes has evolved regional endothermy (Carey, 1982; Carey and Teal, 1966, 1969; Carey *et al*., 1981; Wegner *et al*., 2015). In tunas, and in sharks of the families Lamnidae and Alopiidae, vascular countercurrent heat exchangers conserve metabolically produced heat and maintain elevated temperatures in internalized red (i.e. slow-twitch aerobic) swimming muscles and (in some species) also in the viscera, eyes and brain (Carey and Teal, 1966; Linthicum and Carey, 1972; Block *et al*., 1993; Block and Finnerty, 1994; Graham and Dickson, 2001; Patterson *et al*., 2011). In contrast, billfishes have evolved cranial, but not swimming muscle, endothermy (Carey, 1982; Block and Finnerty, 1994). Regional endothermy affects numerous biological characteristics of relevance to fisheries, including high somatic and gonadal growth rates (Dickson, 1995; Brill, 1996). However, energetic benefits of cold tolerance and elevated metabolic rates are context dependent; they are likely to be advantageous when quality prey is abundant, but not when such prey is scarce (Madigan *et al*., 2015). Collectively, regional endothermy of pelagic fishes enables geographical and vertical niche expansion and increased access to prey (e.g. Block *et al*., 1993; Dickson, 1995; Lowe *et al*., 2000; Schaefer and Fuller, 2010; Madigan *et al*., 2015).

**Figure 2:**
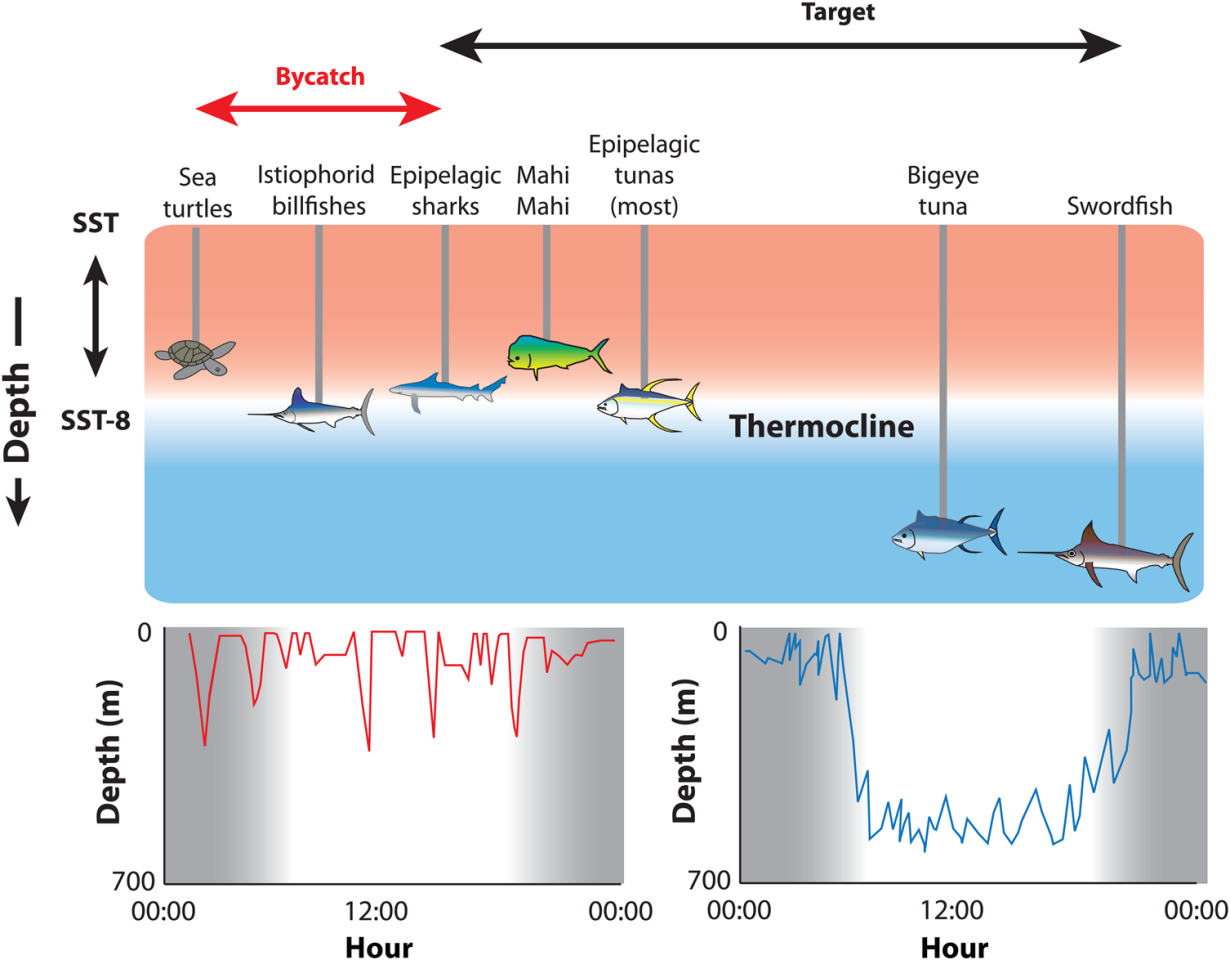
Relationship between ambient temperature and vertical movement patterns of commercially targeted and bycatch species because of the effect of temperature on cardiac function. The influence of temperature on calcium cycling in cardiac tissue limits the movements of a broad taxonomic array of pelagic fishes to the shallow, warm waters above the thermocline (Bernal *et al*., 2009; Galli *et al.*, 2009). The characteristic epipelagic vertical movements (red line, lower lefthand panel) of many billfishes, tunas, and sharks are mostly bounded by the range between sea surface temperature (SST) and 8°C below STT (i.e. SST − 8; Brill *et al*., 1998; Bernal *et al*., 2009). Species that exceed this vertical and thermal range to demonstrate a more characteristic mesopelagic pattern (blue line, lower righthand panel) have cardiovascular adaptations to preserve cardiac function at low temperatures (Galli *et al.*, 2009). Illustrated vertical movement patterns are generalized 24 h periods from the biotelemetry literature (Bernal *et al*., 2009).

Despite their endothermic abilities, temperature conditions with depth still shape the movements, distributions and gear vulnerability of pelagic fishes (Brill and Lutcavage, 2001). There exist two overarching behavioural guilds in pelagic fish vertical thermal niches: a temperature-limited ‘epipelagic’ group bounded by the sea surface temperature and depth of the thermocline (generally 0–200 m), and a thermocline-penetrating ‘mesopelagic’ group capable of extensive vertical movement patterns that follow the diel vertical migrations of organisms of the deep scattering layer, which they exploit as prey (Fig. 2). A litany of electronic tagging studies has revealed that most species of tunas, billfishes, and pelagic sharks, as well as mahi mahi (*Coryphaena hippurus*), largely demonstrate the epipelagic pattern, limiting the majority of their vertical movements to the upper 8°C of the water column between sea surface temperature and the thermocline (reviewed by Brill, 1996; Brill and Lutcavage, 2001; Bernal *et al*., 2009; Braun *et al*., 2015). In contrast, bigeye tuna, swordfish and bigeye thresher sharks (*Alopias superciliosus*) demonstrate the mesopelagic pattern, inhabiting much deeper and cooler waters below the thermocline for most of the day and ascending at night (Bernal *et al*., 2009). Why is a broad taxonomic range of epipelagic-guild endothermic fishes temperature limited, and how do mesopelagic-guild fishes avoid these constraints in spite of phylogeny? There is a simple answer to the first half of the question: the heart lies outside of the influence of countercurrent heat exchangers in all species and thus immediately reflects changes in ambient temperature (Galli *et al*., 2011). The keys to understanding the depth patterns of these two thermal guilds lie in the effects of temperature on cardiac function.

Temperature control of cardiac function predicts species-specific vertical movement patterns in a wide taxonomic range of pelagic fishes. Mechanistic understanding of cardiac thermal niches in pelagic fishes is based largely on experiments conducted on tunas, with analogies extended to billfishes and some pelagic sharks (Brill *et al*., 1998; Weng *et al*., 2005; Bernal *et al*., 2009). In the epipelagic guild, reductions in cardiac calcium cycling impede excitation–contraction coupling in cardiac myocytes when instantaneous temperature changes exceed ∼8°C (Galli *et al*., 2009; Shiels *et al*., 2011). Epipelagic tunas also have extremely limited abilities to compensate for temperature-induced bradycardia by increasing stroke volume (i.e. the volume of blood pumped per heart beat); therefore, cardiac output decreases with heart rate when fish move into cooler waters (Brill *et al*., 1998). Accordingly, most forays to depth by the epipelagic guild must be brief and generally above the thermocline. Cardiac muscle must thereafter be warmed, resulting in a vertical movement pattern reminiscent of an air-breathing vertebrate, in which vertical excursions are followed by surface ‘recovery’ periods (Fig. 2; Horodysky *et al*., 2007). Body size and consequent thermal inertia may influence the variation in vertical movements and ambient temperatures seen among individuals and between species (Goldman *et al*., 2004; Horodysky *et al*., 2007). Deeper-dwelling bigeye tuna, swordfish, and bigeye thresher sharks compensate for life histories expressed in cooler, low-oxygen subthermocline waters via increased lipid stores as insulation and specific adaptations in cardiorespiratory physiology (e.g. Brill *et al*., 1998; Lowe *et al*., 2000; Blank *et al*., 2004; Bernal *et al*., 2009; Galli *et al*., 2009). These fishes forage extensively below the mixed layer by maintaining cardiac function via greater capacity for calcium cycling in the sarcoplasmic reticulum at reduced temperatures (Landeira-Fernandez *et al*., 2004, 2012; Bernal *et al*., 2009; Galli *et al*., 2009). In summary, a long history of electronic tagging studies demonstrates that daily vertical movements and thermal ranges of large pelagic fishes are species specific and thermal guild specific, and physiological experiments demonstrate the specific mechanistic underpinnings of these behaviours. The collective interdisciplinary synthesis (exemplified by Brill *et al*., 1999, 2005; Weng *et al.,* 2005; Galli *et al*., 2009) encapsulates the physical and physiological bounds of fundamental thermal niches, habitats, and vertical movement behaviours in these fishes.

**Figure 3:**
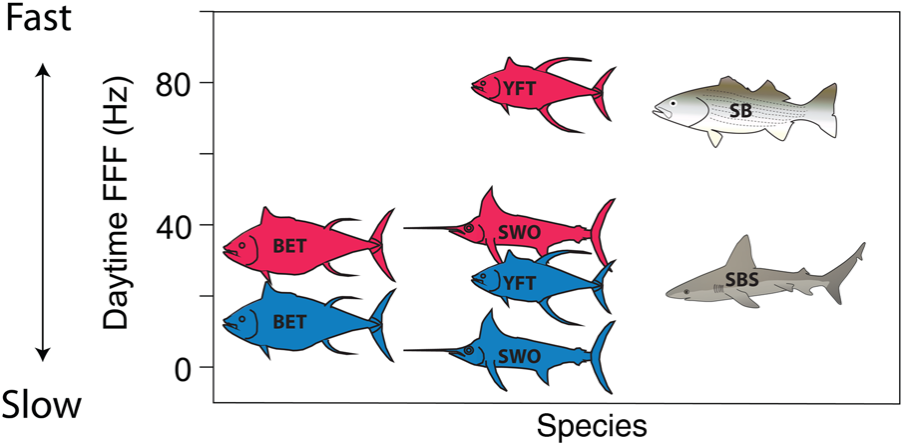
Comparison of temperature effects on temporal resolution (flicker fusion frequency, FFF) in pelagic fishes. Data for swordfishes (SWO), bigeye tuna (BET), and yellowfin tuna (YFT) are from Fritsches *et al*. (2005). Blue fish symbols represent data in ambient conditions at depth, whereas red fish symbols represent temporal resolution in conditions of cranial endothermy. Results for pelagic fishes are compared with coastal pelagic species including striped bass (SB; Horodysky *et al*., 2010) and sandbar sharks (SBS; Kalinoski *et al*., 2014).

Beyond cardiac performance, there is also a sensory advantage to the endothermy of pelagic fishes that allows for vertical as well as latitudinal niche expansion. Cranial endothermy has evolved by convergence in lamnid and alopiid sharks, billfishes, tunas, butterfly mackerel (*Gasterochisma melampus*), and opah (*Lampris guttatus*), making it arguably the most widespread form of regional endothermy in fishes (Runcie *et al*., 2009). The energetic requirements and advantages underlying both the evolution and the maintenance of endothermy are notable. Neural and ocular endothermy expands the thermal niche in the following two ways: (i) it buffers the central nervous system from rapid changes in ambient temperature, allowing the maintenance of neural function; and (ii) it improves temporal resolution and the detection of rapid motion 10-fold, enhancing the ability to track fast-moving prey relative to unwarmed eyes (Fig. 3; Fritsches *et al*., 2005). For example, in swordfish that may traverse a temperature gradient of 20°C or more in minutes, the highly specialized extraocular muscle heater and retial system can warm the eyes and brain to 10–15°C above ambient (Carey, 1982). Similar mechanisms operate in billfishes that share the neural ocular heater mechanism (Carey, 1982; Block, 1986) and, to some degree, in the whole-body endothermic tunas and pelagic sharks (Block and Carey, 1985). In the case of swordfish and istiophorid billfishes, thermal niche expansion was enabled simply by the evolution of a neural heater to compensate for the extreme temperature sensitivity of the retina (Fritsches *et al*., 2005); in tunas and endothermic sharks, thermal niche expansion required whole-body endothermy. Although warmer retinal temperature increases thermal noise, which decreases absolute sensitivity in dim light (Aho *et al*., 1988), neural warming nonetheless provides the large, fast, and sensitive eyes of billfishes with a crucial advantage for detecting, pursuing, and capturing their fast-moving prey in the cold, dim waters in which they hunt (Fritsches *et al*., 2005). Feeding motivation may thus be high in dim, cold, oxygen-poor depths even though some of these fishes (i.e. the epipelagic guild) may not spend the majority of time there; an inference that is counter to applications of HBS assumptions that weigh feeding motivation proportionally to time spent at depth.

Dissolved oxygen also has a substantial influence on the behaviours and distributions of high-oxygen-demand pelagic fishes, increasing the catchability of epipelagic guild fishes by surface fishing gear by constraining available predator and prey habitat to a narrow strip of shallow normoxic surface waters underlain by hypoxic regions (Prince and Goodyear, 2006; Prince *et al*., 2010). Regions of relatively low dissolved oxygen occur across much of the equatorial Atlantic and eastern tropical Pacific and are affected by depth, temperature, productivity, salinity and upwelling (Stramma *et al*., 2008). Their volume, extent, and severity are expected to increase with climate change (Stramma *et al*., 2012). Oxygen is a limiting factor that constrains maximal metabolic rates and metabolic scope of some tunas, and by analogy, billfishes and sharks (Fry, 1947; Bushnell *et al*., 1990). Although vertical movements of epipelagic guild tunas are limited by dissolved oxygen < 3.5 ml^−1^, deeper-dwelling bigeye tuna are tolerant of low ambient oxygen levels, routinely inhabiting waters with dissolved oxygen ∼1 ml^−1^ (Lowe *et al*., 2000). This difference lies in the significantly higher blood O_2_ affinity of bigeye tuna relative to yellowfin tuna, skipjack tuna, and kawakawa (*Euthynnus affinis*) and in potential differences in gill structure that allow mesopelagic guild fishes to extract more oxygen from their oxygen-poor habitat (Bushnell and Brill, 1991, 1992; Lowe *et al*., 2000; Wegner *et al*., 2010). The oxygen requirements of istiophorids are poorly known, but experiments with stressed juvenile sailfish (Idrisi *et al*., 2002) suggest that billfishes have high oxygen requirements typical of tropical epipelagic tunas and are likely to experience hypoxia-based limitation of vertical movements (Brill, 1996; Prince and Goodyear, 2006). Collectively, consideration of temperature, but omission of oxygen, may compromise the habitat standardizations of CPUE trends used by some assessment methods (Bigelow *et al*., 2002).

## Bycatch reduction: keeping gear away from non-target species and vice versa

Bycatch reduction involves two contrasting techniques: (i) keeping the fishing gear away from the bycatch species (i.e. time–area closures, gear deployment strategies); and (ii) keeping bycatch species away from the fishing gear (i.e. making the gear less attractive). The latter bycatch-reduction technologies (i.e. repulsive devices, alternative baits) represent an applied interdisciplinary forum for collaboration. Species-specific sensory insights can be used to enhance the performance or attractiveness of gear for target species (i.e. improving target catchability), a concept that warrants further development. However, although this approach may change target:non-target catch ratios, it is unlikely to change the magnitude of discards or bycatch. Thus, it is an ineffective conservation strategy for commercial pelagic longline, gill net, and purse seine fisheries that interact with protected billfishes and sea turtles. Overly conservative management measures that function by keeping gear away from the bycatch species (e.g. time–area closures) can be economically undesirable. A more economically desirable alternative involves improving selectivity by keeping the non-target species away from fishing gear.

An integrated approach to making gear less attractive to non-target catch requires exploitable differences in the sensory biology and/or behaviours of target and non-target species that allow gear modifications to deter the latter but not the former, lest target catches decline (Southwood *et al*., 2008; Brill *et al*., 2009; Wang *et al*., 2010). Given the broad taxonomic diversity of bycatch (spanning primitive and advanced fishes, reptiles, and birds), it is unlikely that a single solution will reduce all bycatch while simultaneously increasing (or at least not significantly decreasing) the target catch. Rather, a series of strategies is likely to be required for different species, regions, and oceanographic conditions (Hall, 1996). Understanding the sensory abilities of fisheries resources (e.g. Brill *et al*., 2005; Horodysky *et al*., 2008a,b) and bycatch species (e.g. Fritsches *et al*., 2000; Hart and Collin, 2015) is the critical first step to developing a library of potential technologies (Erickson and Berkeley, 2008; Southwood *et al*., 2008; Stroud *et al*., 2013; O'Connell *et al*., 2014a,b; Martin and Crawford, 2015). There are surprisingly few sensory data for many pelagic species (particularly fishes; Brill *et al*., 2005), perhaps owing to the difficulties of their capture and captive maintenance (Box 1).

Once baseline sensory data are obtained and exported to studies of field-based performance of deterrent and attractant stimuli, the efficacy of bycatch technologies can be iteratively tested with field gear modification trials that generate new hypotheses that can then be addressed in the laboratory (e.g. Mooney *et al*., 2007; Brill *et al*., 2009; Hutchinson *et al*., 2012). Effective deterrents, attractants and bait alternatives must have the following attributes: (i) easy and safe to use; (ii) affordable and exportable on a commercial scale; (iii) functional over a wide range of environmental conditions; and (iv) effective for their intended use with minimal reduction in the catch (Brill *et al*., 2009).

To date, the interdisciplinary development and testing of sensory-based bycatch-reduction strategies in pelagic fisheries has spanned the auditory, chemoreceptive (olfactory and gustatory), visual, and electroreceptive senses (reviewed by Southwood *et al*., 2008; Stroud *et al*., 2013). Fishes, marine mammals and sea turtles are all sensitive to low-frequency acoustic signals, which may have an initial deterrent effect on target and non-target catch but may lead to habituation (Moein Bartol and Musick, 2003; Southwood *et al*., 2008; Hart and Collin, 2015). Although a litany of chemicals, including natural defensive compounds, alkaloids, and pungent and bitter substances have been assessed, an effective chemical deterrent for turtle, seabird, and billfish bycatch awaits identification (Southwood *et al*., 2008). Sea turtle bycatch in gill nets may be reduced via visual means, including Plexiglass shark shapes, although with corresponding reductions in target catch, or by the illumination of the net with LED lights or chemical light sticks, which do not affect target catch (Wang *et al*., 2010). Colouring squid baits blue can reduce seabird bycatch (Cocking *et al*., 2008) but not that of all sea turtles (Swimmer *et al*., 2005). Despite dramatic differences in visual spectral sensitivity between mesoplagic-guild and epipelagic-guild fishes, bait colour does not seem to reduce bycatch of fishes or sharks (Brill *et al*., 2005; Southwood *et al*., 2008). Electropositive metals, magnets and semiochemical repellents all show some potential in reducing shark bycatch, but results appear to be temperature and species specific and may be overridden by social cues when fishes are at high density (Stoner and Kaimmer, 2008; Brill *et al*., 2009; Robbins *et al*., 2011; Hutchinson *et al*., 2012; Godina *et al*., 2013; O'Connell *et al*., 2014b). In the energy-depauperate pelagic environment, visual information may override chemosensory and/or electroreceptive input during predatory choices (Southwood *et al*., 2008; Wang *et al*., 2010; Hutchinson *et al*., 2012). Collectively, sensory bycatch studies show some successes in their stated objectives and have evaluated and eliminated other candidate technologies, providing direction for future avenues of research. Sensory-based bycatch research therefore remains a growth area for collaboration between the disciplines of physiology and fisheries science in the interest of conservation biology (Molina and Cooke, 2012; Jordan *et al*., 2013; Hart and Collin, 2015).

## Enhancing survival following release

Sharks, tunas and billfishes occur in extensive commercial and recreational fisheries throughout the world as both target and non-target species. As bycatch cannot be avoided completely, minimizing the impact of fishing gear on the non-target catch is a critical research area. Over the past 30 years, changes in management regulations and increased conservation awareness have resulted in increased live release of pelagic fishes caught by commercial and recreational gears. In North America, for example, increasing proportions of recreationally caught istiophorid billfishes (Goodyear and Prince, 2003), bluefin tuna *Thunnus thynnus* (Stokesbury *et al*., 2011; Marcek and Graves, 2014) and sharks (Sepulveda *et al*., 2015) are released annually, as are all Atlantic billfishes caught by US commercial fishers (Graves and Horodysky, 2015). Internationally, the member nations of the International Commission for the Conservation of Atlantic Tunas (ICCAT) adopted a measure in 2000 requiring mandatory live release of blue and white marlin (*Makaira nigricans* and *Kajikia albida*, respectively) caught in the pelagic longline and purse seine fisheries (Graves and Horodysky, 2015).

The estimation of post-release mortality is a natural collaboration of physiologists and fisheries ecologists. Research into the survival of released fish has identified key predictors of mortality and assessed gear modifications and handling practices that reduce it (Cooke and Schramm, 2007). Standard methods for assessing post-release mortality in fishes, often including confinement (Muoneke and Childress, 1994), are simply not applicable to pelagic fishes given their size, their generally poor success in captivity, and their diversity of complex physiological demands. Efforts to investigate the physical and physiological effects of capture on large pelagic species must therefore include novel techniques used across diverse phylogenetic groups (Skomal, 2007).

The evaluation of the post-release survival of pelagic fishes has been made possible by advances in electronic tagging technologies (e.g. Donaldson *et al*., 2008; Graves *et al*., 2012; Hutchinson *et al*., 2012; Poisson *et al*., 2014; Graves and Horodysky, 2015). Initial studies of the post-release survival of pelagic fishes sought to demonstrate the efficacy of pop-up satellite archival tag technology, then to generate preliminary estimates of post-release survival for assessment purposes and pelagic fisheries management (e.g. Graves *et al*., 2002; Kerstetter *et al*., 2003; Horodysky and Graves, 2005). Investigations of terminal gear configurations (e.g. Prince *et al*., 2002; Domeier *et al*., 2003; Horodysky and Graves, 2005; Graves and Horodysky, 2008; Graves and Horodysky, 2010; Afonso *et al*., 2011) and handling procedures (e.g. Bromhead *et al*., 2012; Schlenker, 2014; Schlenker *et al*., in press) eventually came to include collaborative studies among fishers, physiologists, and fisheries biologists directed at predicting, quantifying, and applying the emerging synthesis in the interest of conservation (Moyes *et al*., 2006; Musyl *et al*., 2011a, 2011b; Gallagher *et al*., 2014).

The change in terminal gear in commercial and recreational fisheries from straight-shank J hooks to circle hooks decreased rates of post-release mortality in many pelagic fishes and other vertebrate bycatch species (Read, 2007; Campana *et al*., 2009; Povano *et al*., 2009; Graves *et al*., 2012; Swimmer *et al*., 2014). In recreational fisheries for white marlin, circle hooks have significantly reduced deep-hooking, hook-induced trauma, and mortality, resulting in management measures requiring their use with natural baits in US fishing tournaments (Horodysky and Graves, 2005; Graves and Horodysky, 2008; Graves and Horodysky, 2015). Although not universally the case, similar effects of circle hook performance on survival are generally seen with tunas, istiophorid billfishes, elasmobranchs, and sea turtles in commercial pelagic longline gear (Kerstetter *et al*. 2007; Campana *et al*., 2009; Povano *et al*., 2009; Afonso *et al*., 2011; Pacheco *et al*., 2011; Graves *et al*., 2012; Swimmer *et al*., 2014) and in recreationally caught tuna (Stokesbury *et al*., 2011). Results from recreational fisheries targeting striped and blue marlin are consistent but more subtle, presumably because of differences in fishing techniques and behavioural differences in how these fishes attack baits (Domeier *et al*., 2003; Graves and Horodysky, 2010). The collective synthesis is that by virtue of differences in shape, circle hooks are more likely to hook fish and other vertebrates in the jaw than straight-shank J hooks and are thus less likely to cause immediate or delayed internal trauma and physiological wasting (Borucinska *et al*., 2002; Cooke and Suski, 2004; Swimmer *et al*., 2014; Graves and Horodysky, 2015). Hook-induced injuries and consequences of capture may range from sublethal (but with fitness repercussions) to lethal, and mortality may be immediate or occur hours to weeks after the capture event (Wilson *et al*., 2014). Sublethal effects of hooking and capture have thus far received considerably less attention because of the logistic challenges associated with doing so for this group of animals.

To assess mortalities associated with capture stress synoptically, researchers must look beyond mortality that immediately follows the capture event to examine the cumulative impacts of physical trauma and physiological stress (Skomal, 2007). Capture stresses involve: (i) the physical trauma of hooking, fighting, and handling; and (ii) the physiological stresses of catch, exhaustive exercise, handling, and recovery (Cooke and Suski, 2005; Skomal, 2007; Wilson *et al*., 2014). Released fishes may experience fitness consequences ranging from perturbations in blood acid–base balance and ion levels (e.g. Skomal and Bernal, 2010; Marshall *et al*., 2012; Kneebone *et al*., 2013) to physical injury (Pranovi *et al*., 2001) to mortality (Kaiser and Spencer, 1995). These fitness consequences result from the following factors: (i) interactions with the fishing gear itself (which may result in catch or escapement); (ii) capture by the fishing gear; (iii) landing onto a vessel; (iv) retention on deck during catch-sorting operations; or (v) entanglement in materials used to construct fish aggregation devices (Filmalter *et al*., 2013; Ingolfsson *et al*., 2007; Giomi *et al*., 2008). Physiological techniques continue to provide insights into the effects of interactions with fishing gear, capture and subsequent release (e.g. Moyes *et al*., 2006; Skomal, 2007; Skomal and Bernal 2010; Cooke *et al*., 2012a; Marshall *et al*., 2012; Hutchinson *et al*., 2015), improving the welfare of fishes and other vertebrates released from commercial gillnets, purse seines, trawls, and longlines (Farrell *et al*., 2001; Marçalo *et al*., 2006; Mandelman and Farrington, 2007; Marshall *et al*., 2012) as well as recreational fishing gear (Cooke and Schramm, 2007). Indeed, such information is increasingly being used in various certification programmes (e.g. Marine Stewardship Council) to evaluate the sustainability of a given fishery.

Physiological studies of pelagic fishes following capture have largely focused on perturbations to homeostasis reflected in blood or muscle chemistry (Wells and Davie, 1985; Mandelman and Skomal, 2009; Skomal and Bernal, 2010; Marshall *et al.*, 2012; Schlenker, 2014). Skomal and Chase (2002) and Skomal (2006) quantified changes in blood acid–base status, metabolites, electrolytes, and proteins in several species of sharks, tunas, and marlin after capture and release on recreational fishing gear, finding significant interspecific differences in the magnitude and source of these disturbances, with greatest disruption in the tunas. Skipjack tuna also display pronounced acidosis after induced exhaustive exercise, but have extremely rapid lactate turnover (∼1 h), rivalling mammals (Perry *et al*., 1985; Wells and Davie, 1985; Weber *et al*., 1986, Wells *et al*., 1986; Arthur *et al*. 1992). This outcome stands in stark contrast to results observed in sharks that suggest lengthy perturbations for up to 24 h (reviewed by Skomal, 2007). Moyes *et al*. (2006) delineated five variables linked to strenuous muscular activity and resulting physiological stress (acidosis) and tissue damage (myopathy) that distinguished surviving from moribund longline-caught sharks: Mg^2+^, lactate, *Hsp70* mRNA, K^+^ and Ca^2+^.

Novel applications combine field-based tagging and laboratory approaches in the study of fish post-release ecophysiology, and iteratively verify assays with field-based measures and telemetric technologies. In a study of 11 shark species, Marshall *et al*. (2012) documented species-, family- and ecology-specific responses in plasma electrolyte levels (Na^+^, Cl^−^, Mg^2+^, Ca^2+^ and K^+^), metabolites (glucose and lactate), blood haematocrit, and heat shock proteins following longline capture; Marshall (2015) verified survival with satellite tags in a subset of species. A similar biochemical and satellite tagging approach was used for white marlin released from recreational fishing gear by Schlenker (2014), who found elevated K^+^ concentrations to be a predictor of mortality. Taken together, the results of Marshall *et al*. (2012) and Schlenker (2014) suggest that hyperkalaemia deserves further investigation as a mechanistic predictor of mortality.

Many studies investigating the effects of angling on the post-release survival of pelagic fishes overlook the fundamental relationships between drag, metabolism, and endurance, assuming that physiological perturbations are proportional to the duration of exertion. Significant positive relationships between stress and the duration of the capture event have been demonstrated in a diverse group of fishes (Skomal, 2006; Suski *et al*., 2007; Cooke *et al*., 2008; Heberer *et al*., 2010; Gallagher *et al*., 2014). The consideration of fight time as a mechanistic variable has understandable appeal because it can lead to practical management policy outcomes that provide best-practice angling guidelines. Unfortunately, simple correlation of fight time and stress overlooks the fact that hooked fishes can exert maximal fighting effort for only fractions of a minute because of the steep inverse relationship between endurance and swimming performance (Horodysky *et al*., 2015). Fishes can therefore fight maximally for a very short time, fight minimally for a long time or fight maximally in bursts followed by rest periods for an intermediate time. Although fight time may serve as an adequate stress proxy for the first two fight strategies, the burst–rest strategy confounds the utility of fight time as a predictor. This is likely to be the case in many pelagic fishes that switch between blistering runs, acrobatic aerial displays, and sounding and circling behaviours in deeper, cooler waters during capture on recreational fishing gear. Correlating blood perturbations with cumulative ‘fight time’ without considering the changing nature of the intensity of activity risks potentially biased or confounded outcomes (Horodysky *et al*., 2015). Triaxial accelerometers present novel options to quantify changes in activity of angled fish (Kneebone *et al*., 2013), but to our knowledge this technology has not yet been used with pelagic fishes.

Collectively, while assessing post-release mortality in pelagic fishes is difficult and requires multiple laboratory, telemetric, field-based approaches that quantify the extent of physical damage and the level of physiological disruption, such data are critically needed for the conservation of pelagic species. Integrations of the disciplines of molecular biology, telemetry, behaviour, and population modelling have the potential to provide more robust inferences (Davie and Kopf, 2006; Moyes *et al*. 2006; Musyl *et al*., 2011a; Cooke *et al*., 2013) to inform fisheries management and educate fishers about angling and handling best practices (Cooke and Schramm, 2007). Specifically, physiological techniques can be used to develop handling procedures that reduce sublethal stresses and/or avoid lethal outcomes (Cooke *et al*., 2002; Suski *et al*., 2007; Mandelman and Skomal, 2009), and may be combined with quantitative techniques to assess the bioenergetics and other fitness consequences of capture (Meka and Margraf, 2007; Musyl *et al*., 2011a). We view this as a major growth area for the interdisciplinary collaboration of fisheries science and physiology.

## Conclusions and future directions

The conservation physiology of pelagic fishes provides both formidable challenges and exciting interdisciplinary opportunities. Pelagic fishes support some of the most important fisheries in the world, and while target species are fished near capacity, many non-target species are overfished. There is thus an exigent need to understand better the mechanistic relationships of pelagic fishes to their environment and fishing gear, the associated feedback mechanisms from the cellular to the population level, and the effects of anthropogenic stressors (i.e. fishers' behaviour) across this continuum. Pelagic habitats and their fishes are remote frontiers for mechanistic research, because access to them is both logistically difficult and expensive. Many of these species cannot tolerate captivity. Despite these challenges, new technologies and interdisciplinary collaborations are inspiring new research directions, disciplinary Rubicons are being crossed, and insights are being obtained from the cellular to the population level. Perhaps the greatest collaborative successes in pelagic fish conservation physiology have resulted from studies of fish–environment relationships, bycatch reduction, and assessment of the factors that affect rates of post-release survival. The iterative testing by one discipline of hypotheses generated by the other is beginning to enclose the fundamental−applied science continuum, leading to the development of robust mechanistic insights that lead to informed management outcomes.

**Figure 4:**
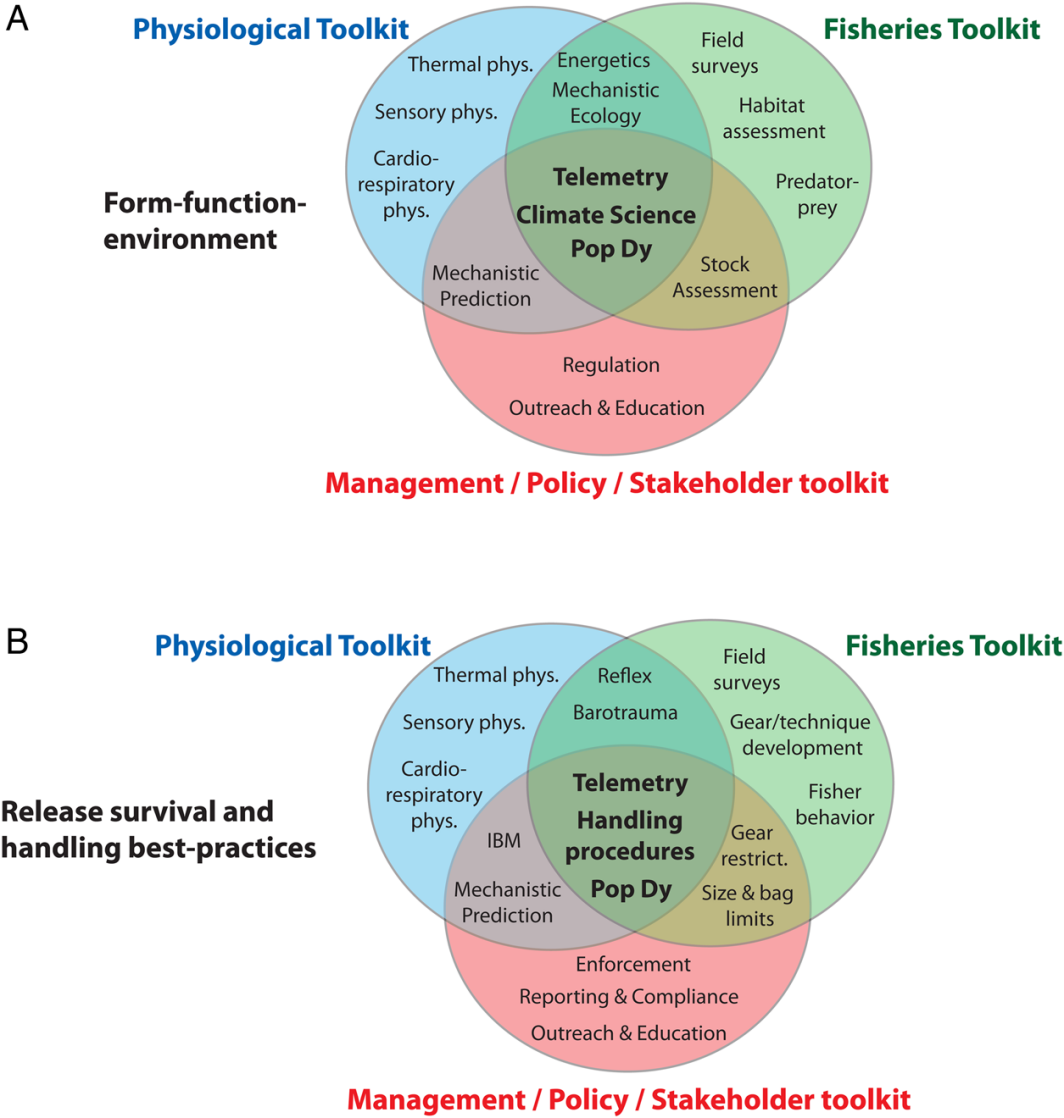
Venn diagram of the potential toolkits provided to studies of form–function–environment relationships (**A**) and release survival best practices (**B**) by both the disciplinary-specific contributions and interdisciplinary interactions of the fields of fish physiology (blue) and fisheries science (green) when combined with the management, policy, and stakeholder arena (red).

The future of pelagic fish conservation physiology lies in interdisciplinary interactions of the perspectives and toolkits possessed by the disparate disciplines of physiology and fisheries science and their integration with the needs of fisheries managers and stakeholders (Fig. 4). Overcoming logistical challenges to obtain physiological data will help to parameterize stock assessment models to improve mechanistic prediction of populations, responses to environmental change, and the effects of management plans and harvest strategies, ultimately providing better tools to guide management decisions and interventions (Cooke *et al*., 2014a). In the interests of conservation and stewardship, physiology must be further integrated into pelagic fisheries management and conservation (Cooke and Suski, 2008; Cooke and O'Connor, 2010).

We believe, as have others (Cooke and O'Connor, 2010), that fish physiologists, fisheries scientists, and resource managers should further their collaborations to identify, plan, and evaluate future research directions and their products, and this is especially so in the frontier field of pelagic fish conservation physiology. As evidenced in the examples provided above, physiology offers a suite of tools to establish cause-and-effect relationships, provide baseline background data, and suggest and monitor the efficacy of management strategies (Wikelski and Cooke, 2006). Given that protecting all habitats and species is unrealistic, physiological tools can identify critical habitats of functional and temporal importance and the individual-level benefits associated with management recommendations (e.g. improved fitness; Cooke and Suski, 2008; Cooke *et al*., 2013). Furthermore, the relationship between physiology and the environment can inform management action via the incorporation of such data in population models, individual-based models, species distribution models, and mass- or energy-balance models (Metcalfe *et al*., 2012). Ultimately, the effective integration of physiological approaches in the synoptic management and sustained use of pelagic fish stocks requires interdisciplinary collaborations between scientists in several subdisciplines, fisheries managers and regional fisheries management agencies, and fisheries stakeholders.

## Funding

A.Z.H. acknowledges support from the NOAA Living Marine Resources Cooperative Science Center and NSF Educational Partnership in Climate Change and Sustainability. S.J.C. is supported by NSERC, Ocean Tracking Network Canada, and the Canada Research Chairs Program.
